# RNA: methods and protocols — a new series

**DOI:** 10.1186/1758-907X-3-7

**Published:** 2012-06-07

**Authors:** Phillip D Zamore

**Affiliations:** 1 Howard Hughes Medical Institute and Department of Biochemistry and Molecular Pharmacology, University of Massachusetts Medical School, 364 Plantation Street, Worcester, MA, 01605, USA

## Abstract

This month, *Silence* launches a new series on methods and protocols to study silencing pathways and analyze nucleic acids and proteins.

## **Editorial**

This month, *Silence* launches a new series on methods and protocols to study silencing pathways and analyze nucleic acids and proteins. The first article in this series, from Xuemei Chen and coworkers [1], reports the development of a novel reporter system to enable forward genetic screens for transcriptional silencing in plants. This article exemplifies the type of methods we plan to highlight: robust, quantitative tools and protocols that make it easier to study silencing phenomena in particular, but also the regulation of gene expression more broadly. We welcome not only articles describing novel techniques and tools, but also modifications and improvements that advance existing methods beyond their current use.

As *Silence* provides open access to all its articles, the methods and protocols published in the journal are freely available to all. New methods, for example, to detect RNA molecules — in eukaryotes or in prokaryotes, whether short or long, individually or genome-wide — published in this series will therefore reach a wide audience and enable new and better research. It is particularly gratifying to inaugurate the series with a report detailing the construction, validation, and use of a new tool for genetic screening, since the known silencing phenomena in bacteria, plants, fungi, and animals were all discovered by classical forward genetics. We look forward to publishing other genetic tools and screening methods, as well as techniques for mapping novel alleles. The study of gene regulation desperately needs new protocols for measuring and sequencing RNA that decrease data bias, require fewer cells for reliable detection, accelerate the time invested in sample preparation, and reduce assay cost. Genome sequencing and viral discovery are increasingly affordable, but improvements in assays and analysis are clearly needed. High throughput methods currently enable the detection of nearly all the RNA or DNA sequences bound to a specific protein, but the techniques and analytical tools clearly can stand improvement. *Silence* looks forward to reporting advances in such established methods alongside descriptions of entirely novel tools and algorithms.

To submit your manuscript, please use our online submission system and indicate in your cover letter that you would like it to be considered for the series; alternatively, send a pre-submission enquiry to editorial@silencejournal.com.
